# Collecting and Delivering Fattened Pigs to the Abattoir

**DOI:** 10.3390/ani14111608

**Published:** 2024-05-29

**Authors:** Lluís Miquel Plà-Aragonés, Yun Bao, Pol Llagostera, Angel Juan, Javier Panadero

**Affiliations:** 1 Department of Mathematics, University of Lleida, 25001 Lleida, Spain; yb10@alumnes.udl.cat (Y.B.); pol.llagostera@udl.cat (P.L.); 2 Agrotecnio CERCA Center, 25198 Lleida, Spain; 3 Research Center on Production Management and Engineering, Universitat Politècnica de València, 03801 Alcoy, Spain; ajuanp@upv.es; 4 Department of Computer Architecture & Operating Systems, Universitat Autònoma de Barcelona, 08193 Bellaterra, Spain; javier.panadero@uab.cat

**Keywords:** fattened pigs, abattoir, vertical integration, PJS heuristic, team orienteering problem

## Abstract

**Simple Summary:**

This article discusses how to plan the transport of fattened pigs from farms to the abattoir efficiently when the farms are coordinated with the abattoir operation. In contrast to the papers published to date that have been concerned with the farmer’s profit, this paper adopts the slaughterhouse perspective. Coordination with growers should help to better plan the collection of pigs by establishing the best routes for the trucks to ensure the optimal functioning of the abattoir. Starting from a real scenario, the study delves into a complex Team Orienteering Problem, taking into account factors such as stochastic production and maximum truck workload. The paper adopts the PJS heuristic and compares it with exact methods, revealing the computational infeasibility of the latter. Through scenario analysis, the study unveils insights into practical solutions, highlighting a positive relationship between the exploration of alternative routes, the number of trips, transport costs, and maximum reward. In particular, greater variability in the number of pigs to be collected presents opportunities for a more efficient mix of loads, especially if one truck can visit at least one more farm. These results offer valuable insights for optimizing the pig transport logistics, potentially improving the profitability and sustainability.

**Abstract:**

In the context of pig farming, this paper addresses the optimization problem of collecting fattened pigs from farms to deliver them to the abattoir. Assuming that the pig sector is organized as a competitive supply chain with narrow profit margins, our aim is to apply analytics to cope with the uncertainty in production costs and revenues. Motivated by a real-life case, the paper analyzes a rich Team Orienteering Problem (TOP) with a homogeneous fleet, stochastic demands, and maximum workload. After describing the problem and reviewing the related literature, we introduce the PJS heuristic. Our approach is first compared with exact methods, which are revealed as computationally unfeasible. Later, a scenario analysis based on a real instance was performed to gain insight into the practical aspects. Our findings demonstrate a positive correlation between the number of alternative routes explored, the number of trips, the transportation cost, and the maximum reward. Regarding the variability in the number of pigs to collect, when a truck can visit more than one farm, better solutions can be found with higher variability since the load can be combined more efficiently.

## 1. Introduction

In countries like Spain, the pig sector plays a key and competitive role [1]. The growth in pig companies and increasing competition have led to increasingly complex decision-making processes, necessitating specialized models to maintain competitiveness [2]. Spain leads Europe in pig production, with a supply chain dominated by vertically integrated companies or cooperatives, while independent individual farmers are becoming scarce. The integration in the pig sector began primarily around feed mills in the 1970s, leading to a concentration of pig production separate from the meat packing plant operations, creating a bottleneck in the Pig Supply Chain (PSC) between the two dominant subsystems: farming production and pork marketing [2]. However, over the last few decades, economies of scale have accelerated the changes globally, further promoting integration [3,4]. Consequently, Spanish pig production has evolved to include meat packing plants, abattoirs, and other connected business activities, such as consulting services, medical products, veterinary services, or engineering offices. In this context, the integrator, whether a private company or cooperative, operates as a PSC. Therefore, while farmers were once the primary on-farm decision-makers, today, decisions primarily rest with the integrators’ headquarters as they coordinate their own PSC, composed of different decision-making units or PSC agents [5,6]. Simultaneously, traditional open market relationships have been replaced by new marketing agreements seeking higher degrees of coordination among the chain agents [7].

In general, the specialization and technical advancements in the pig sector have complicated the decision-making processes, necessitating a holistic view compatible with consumer and societal preferences [2,4,5]. Most models proposed for pig production planning have focused on individual farm perspectives, reflecting the realities in countries where farmers have significant control and autonomy, such as France [8]. However, some models overlook transportation planning [3]. Specifically, the problem of delivering pigs to the abattoir has been explored by various authors. For instance, Pourmoayed et al. [9], Rodríguez-Sánchez et al. [10], and Davoudkhani et al. [8] consider the perspective of individual growers selling pigs to the abattoir. In contrast, few decision models in the literature consider the perspective of the Pig Supply Chain (PSC), which involves managing multiple fattening farms concurrently [4,6]. The focus of a PSC manager is on optimizing the abattoir operations, starting with coordinating fattening farms to slaughter pigs to meet the pork demand rather than optimizing individual farmers’ slaughter times. Thus, the collection of fattened pigs with marketable weight across integrated farms should be planned from the abattoir’s perspective and coordinated by the PSC manager to prevent disruptions and downtime at the meat packing plant. An effective optimization model for PSC coordination must consider coordinating all the fattening farms involved in pig marketing, in addition to the abattoir. Consequently, coordination is typically executed by a fleet of vehicles responsible for collecting and delivering the pigs to the abattoir under the supervision of the PSC manager. The fleet must address the Team Orienteering Problem (TOP) [11,12], determining which vehicle visits which farm to collect the pigs for slaughter. The TOP approach aims to optimize the routing of the trucks that can collect pigs by visiting a subset of farms with different reward values within a limited time frame. The objective is to maximize the total collected reward while considering constraints such as transportation cost, time limitations, and limited truck capacity. Given that farm and abattoir operations are scheduled weekly, the objective of this paper is to propose a TOP model formulation to plan the weekly collection of pigs for delivery to the abattoir. Therefore, the main contributions of the paper are the following:

- Provide a conceptual description of the problem of marketing fattened pigs to the abattoir from the abattoir’s perspective.
- Formulate a Team Orienteering Problem (TOP) and solve it for large instances using an algorithm that extends the Panadero–Juan savings (PJS) heuristic [12].
- Conduct a scenario analysis based on a real-life case to investigate and discuss the main practical implications.

The remainder of the paper is structured as follows: Section 2 presents the state of the art regarding the TOP based on a literature review of the recent papers published in indexed journals. Section 3 describes the real problem from the abattoir’s perspective. In Section 4, methods such as the linear programming formulation and the approximate approach, as well as the data available from a real-life scenario, are presented. Section 5 introduces the results of the scenario analysis and discusses the most relevant findings and limitations of this study. Finally, Section 6 provides the main conclusions and outlines future research directions.

## 2. The Team Orienteering Problem in Agriculture

The Team Orienteering Problem was introduced by Chao et al. [11], who proposed a linear programming model aiming to determine *m* routes starting and ending at specific points through a subset of locations. The goal is to maximize the total reward given a fixed amount of time for each team member. An extensive literature review on this topic, reporting the different variants of this problem, was published by [13]. Despite the TOP not being a novel problem in the field of Operational Research, there are relatively few applications in agriculture. A search on ScienDirect with the terms “Team”, “Orienteering”, “Problem”, and “Agriculture” yields only 45 references, and not all of them are concerned with applying the TOP to agricultural problems. Many of these papers merely mention agriculture as a potential field of application. In fact, only four papers are directly related to agricultural research. Two of them address the optimal spraying tasks in crop protection with multi-Unmanned Aerial Vehicle (UAV) systems [14,15], while the other two focus on farm monitoring, also utilizing UAVs [16,17]. Clearly, the use of UAVs has been the dominant field of application for the TOP model thus far, but there is a noticeable absence of scientific articles concerning livestock production.

## 3. Understanding the Real Problem for the Abattoir

**Figure 1:**
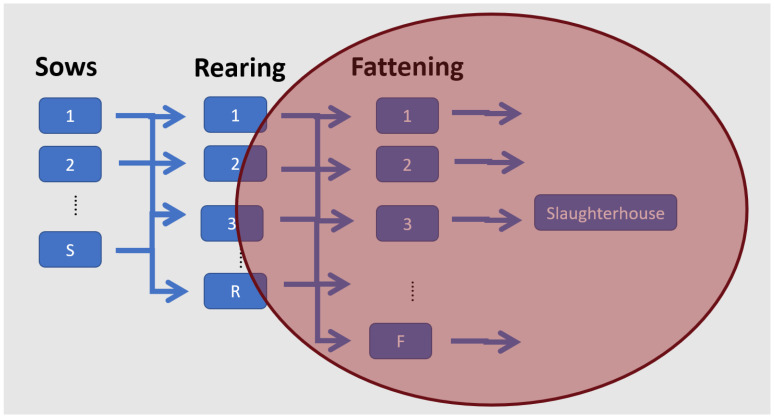
Pig Supply Chain structure and coordination.

The problem addressed in this paper is based on a real-life scenario encountered by PSC managers, who must coordinate the transportation of fattened pigs from various fattening farms to the abattoir (Figure 1). This scenario is a usual one in vertically integrated companies or cooperatives globally [3], including the Spanish pig sector [6]. Each week, the abattoir needs to organize trips to collect the marketable pigs from different fattening farms owned by the integrator. Pig farming encompasses various types of essential farms, as illustrated in Figure 1: sow farms, rearing farms, and growing–fattening farms. However, different combinations of sow-rearing farms, rearing–fattening farms, or farrowing-to-finish farms may coexist under the same integrator. The number of marketable pigs in each fattening farm is estimated by a farm visitor, an individual assisting the PSC manager who estimates pig live weights visually [10]. These estimations are utilized to determine the tentative date for commencing pig deliveries to the abattoir and clearing the farm. Fattening farms adhere to an all-in-all-out (AIAO) management policy, meaning that all pigs enter the farm as a batch, and a new batch can only be introduced once all the pigs from the previous batch have been delivered to the abattoir and the facilities have been cleaned.

Since not all pigs grow at the same rate, the delivery of pigs to the abattoir typically lasts about four weeks between the first and the last load of animals [10,18]. Moreover, as all the fattening farms supply pigs to the same abattoir and belong to the same integrator, the delivery of pigs over time must be balanced throughout the year, and farms house batches of pigs at different growing stages. In a balanced production system, the number of weekly trips to the abattoir depends on the slaughtering capacity. Additionally, the number of trips per truck depends on the size of the available fleet owned by the integrator or abattoir and the distance of farms to cover. Depending on the country, different transportation regulations apply to the welfare of animals during loading, driving, and unloading (e.g., EU Regulation No. 1/2005). This includes the maximum capacity of trucks, minimum room per pig, or maximum distance to cover.

Once the total number of pigs available to be collected on each farm is determined, scheduling load trucks must consider the incompatibility constraints between the workload tasks and ensure that a minimum number of pigs are collected. Incompatibilities between farms can result from housing different breeds or the sanitary status of the herds. Transport routes are planned by the transport manager, abattoir, or PSC manager according to the commercial link between these actors in the PSC. These plans depend on the details of the contract and are not always related to the distance that needs to be covered [19]. The integrator may own a fleet of vehicles for transportation. However, outsourcing is also common in practice, particularly during peak periods. Therefore, the abattoir is served by a team of truck drivers based at the depot. Each week, the team leaves the depot to execute the schedule for that week and returns to unload. The daily schedule for a week is flexible and only needs to fulfill the abattoir demand and balance the workload of drivers. Slaughtering of third-party pigs is also possible to keep the workload at the abattoir balanced over time or to profit from opportunity cost, but priority is afforded to in-house-produced animals. The problem thus involves constructing a schedule to collect pigs from farms so that the weekly abattoir demand is met and the total reward associated with collecting the fattened pigs is maximized. Since a farm can only be visited once a week, it can only belong to one route in the context of a vehicle routing problem. The reward of each farm depends on the live weight of the animals, the lean content, and the bonus agreed upon by farmers with the abattoir [10]. The objective is to maximize the rewards derived from collecting pigs from farms minus the transportation cost. To the best of our knowledge, this problem has never been addressed in the scientific literature and can be viewed as a TOP with additional constraints. In particular, these additional constraints are the following:

- Each vehicle route starts and ends at the abattoir.
- The accumulated quantity of pigs carried by each vehicle does not exceed the total carrying capacity due to animal welfare regulation of the European Union (EU Regulation No. 1/2005).
- There is a maximum time limit for vehicle routes.
- Each farm delivers at most only once by the same vehicle at the same period.
- If the truck capacity is sufficient, a truck visiting a farm must collect all available pigs.

The anticipated outcomes of the proposed approach benefit from an understanding of the context in which farmers and pig companies make decisions, and it is expected that they will facilitate the future deployment of new decision support systems [20]. This is a relevant contribution considering the competitiveness of the sector and the absence of specialized tools to facilitate data-driven decisions.

## 4. Materials and Methods

This section provides a formal description of the problem, introduces the proposed solving approach, and elaborates on a real-life case study.

### 4.1. Mathematical Formulation

The proposed TOP can be defined on a complete directed network. Let $G=(V^{\prime },A)$ be a complete directed graph with vertex $0\in V^{\prime }$ representing the abattoir, where the route starts and ends, while set $V=1\ldots n\subset V^{\prime }$ represents the farms’ locations; set *A* is the arc set. Each vertex i∈V has an associated profit $p_{i}\in R^{+}$ and each arc (i,j)∈A has a travel time $t_{ij}\in R^{+}$ calculated from real distances among farms. The length of a path (measured in time) cannot exceed the predefined time limit $T_{max}$. Every farm (vertex) can be visited at most once. The decision variables are as follows:

- Binary decision variables $x_{ij}^{d}\in \left\{0,1\right\}$, where each $x_{ij}^{d}$ takes the value 1 if farm *j* is visited immediately after farm *i*, with truck *d*, being 0 otherwise.
- Integer decision variables $y_{i}^{d}\in N$, where each $y_{i}^{d}$ takes the value *z* if farm *i* is the $z^{th}$ visit of truck *d*, being 0 otherwise.

Then, the mathematical model can be formulated as

(1) $$\begin{array}{cc} \mathrm{Maximize}\; & \sum_{d\in D}\sum_{(i,j)\in A}u_{j}\cdot x_{ij}^{d} \end{array}$$

(2) $$\begin{array}{cccc} s.t.:\; & x_{ij}^{d}\leq 1 & \; & \forall (i,j)\in A,\forall d\in D \end{array}$$

(3) $$\begin{array}{cccc} \; & y_{i}^{d}-y_{j}^{d}+1\leq \left(1-x_{ij}^{d}\right)\cdot \left|V\right| & \; & \forall i,j\in V,\forall d\in D \end{array}$$

(4) $$\begin{array}{cccc} \; & \sum_{i\in V}x_{ij}^{d}=\sum_{h\in V}x_{jh}^{d} & \; & \forall j\in V,\forall d\in D \end{array}$$

(5) $$\begin{array}{cccc} \; & \sum_{j\in V}x_{0j}^{d}=\sum_{i\in V}x_{i0}^{d}=1 & \; & \forall d\in D \end{array}$$

(6) $$\begin{array}{cccc} \; & \sum_{(i,j)\in A}t_{ij}\cdot x_{ij}^{d}\leq T_{max} & \; & \forall d\in D \end{array}$$

(7) $$\begin{array}{cccc} \; & x_{ij}^{d}\in \left\{0,1\right\} & \; & \forall (i,j)\in A,\forall d\in D \end{array}$$

(8) $$\begin{array}{cccc} \; & y_{i}^{d}\in N & \; & \forall i\in V,\forall d\in D \end{array}$$

Equation (1) represents the objective function to be maximized. Constraint (2) ensures that each farm should be visited by a truck at most once during the entire time horizon. Constraint (3) prevents the formation of subtours. Constraint (4) includes a flow balance constraint, ensuring that any arrival at a farm is compensated by a departure. Constraint (5) dictates that all vehicles must depart from and return to the abattoir (vertex 0). Constraint (6) stipulates that the total travel time of each vehicle should not exceed its threshold. Finally, constraints (7) and (8) define the nature of the $x_{ij}^{d}$ and $y_{i}^{d}$ variables.

### 4.2. Solution Approach

Given the complexity of the model in Equations (1)–(8), obtaining an exact solution within a reasonable computational time is not feasible. Therefore, a heuristic based on the PJS algorithm [12] is proposed. The general steps of the algorithm are detailed in the pseudocode presented in Algorithm 1. The basic idea is to explore the space of solutions iteratively, while retaining the best solution found. The key process of the algorithm is the *merge*() function, which incorporates the PJS heuristic to construct new solutions.
**Algorithm 1** PJS pseudocode adapted to the collection and delivery of pigs to the abattoirData:BR,maxSave,nodes,dMat,elapsedsol←genInitSol(maxSave,nodes,dMat)eff_list←create_eff_list(sol)eff_list←sort(eff_list)**while** $elapsed<T_{max}$ **do**    new_sol←merge(BR,maxSave,nodes,dMat,eff_list)    **if** new_sol>sol **then**        sol←new_sol    **end if****end while**Print the best solution sol

A key aspect of the proposed approach is the generation of the efficient list, eff_list, by the function create_eff_list() (see Algorithm 1). When an edge is created, both its cost and efficiency are computed. Specifically, efficiency is calculated as a linear combination of cost and reward, depending on a parameter α∈[0,1]. Once all edges are created with their corresponding cost and efficiency, they are sorted by efficiency in the eff_list. This ordered list serves as the basis for constructing new solutions by attempting to combine (merge) the most promising edges. Moreover, the selection of elements from the list to build a new solution is randomized at each iteration. This random selection approach allows for better exploration of the solution space and prevents deterministic outcomes that would arise from always selecting elements of the list in the same order.

Models (1)–(8) were implemented in IBM OPL and solved with CPLEX 12.0, while Algorithm 1 was implemented in Python 3.11. Both the model and the algorithm were executed on a 2.10 GHz Intel^®^ Core^TM^i7-1260P with 16 GB of RAM running the Windows 11 Pro operating system. The same time constraint was applied to both methods to obtain a solution. For statistical analysis and plotting, JMP^®^ 17 Pro was utilized.

### 4.3. The Real Instance

We obtained permission from a medium-sized Spanish pig integrator company (kept confidential) to utilize the locations of their various fattening farms and their own abattoir. The abattoir has a slaughtering capacity of 5000 pigs per day, and the integrator operates 186 fattening farms, producing nearly a million pigs annually. The abattoir accepts third-party pigs for slaughter but gives priority to those produced by the same company, providing flexibility in managing their own production. The abattoir is responsible for organizing the collection and transportation of fattened pigs from farms on a weekly basis. The typical truck capacity ranges between 200 and 220 heads, with 200 pigs being the most common capacity currently due to an increase in slaughtering weight in recent years.

**Figure 2:**
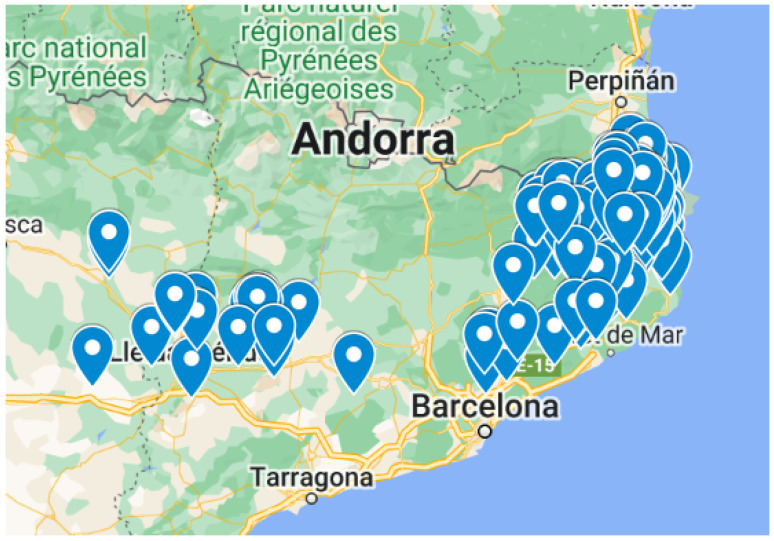
Distribution of farms around a geographical area.

Figure 2 displays the geographical locations of the facilities considered in this study on a map, with the latitude and longitude provided by the company for all farms and the abattoir.

Instead of using a distance matrix, a travel time matrix was built using the coordinates of all the farms and abattoir provided by the integrator. To accomplish this, the URL https://project-osrm.org/ (accessed on 6 December 2023) was utilized. This URL serves as an endpoint for the Open Source Routing Machine (OSRM) service, specifically designed for generating travel time and distance matrices for driving routes. Correct requests to this endpoint generate a JSON response containing a matrix of travel times and distances between multiple pairs of locations based on driving routes. Only travel times were utilized in this study. The reward of each farm is calculated according to [10]. The rest of the production system was not taken into account (e.g., like sow or rearing farms), also leaving aside considerations regarding other production stages.

In the evaluation of different scenarios for the abattoir, several practical considerations were assumed regarding fattening farm capacity and operation:

- All farms are assumed to be operating.
- The full capacity of farms is considered; however, we are only concerned with heads or tails in batch production.
- Welfare and sanitary regulations affecting swine transport [19] are assumed to be correct.
- Third-party transportation is not considered, and fleet size is not constrained.

### 4.4. Scenario Analysis

The scenario analysis was designed over a large set of instances based on the real instance presented in Section 4.3. The parameters used to simulate different scenarios considered three important dimensions: (i) the number of marketable pigs; (ii) the homogeneity or variability of available pigs; and (iii) the capacity of available trucks. In order to balance costs, tuning of the alpha value involved in the PJS heuristic was performed.

A total of 10 (average number of marketable pigs) × 5 (different homogeneity levels or standard deviations) × 3 (maximum truckload) = 150 scenarios were simulated. For this purpose, each scenario was recorded in a coded file named mXsdYZZZ, where *X* refers to the average number of pigs to collect, *Y* is the standard deviation, and ZZZ is the maximum load of trucks. The average values and standard deviation values were used to randomly generate normal observations for the number of pigs in each fattening farm. The same values were maintained for the different truck capacities considered (parameters ZZZ). Any random value outside the range [0,ZZZ] was replaced by its corresponding extreme value, i.e., 0 or ZZZ.

The outputs recorded in each scenario included the following: (i) the best solution number with the corresponding best cost; (ii) the reward obtained; and (iii) the number of routes generated. For further inspection and analysis, a more detailed file was recorded, providing specific routes within the solution. Additionally, auxiliary calculations were performed to derive supplementary variables, providing further insights into the analysis of results. These calculations are detailed in Table 1.

## 5. Results and Discussion

This section provides the results obtained and the corresponding statistical analysis.

### 5.1. Base Case: Preliminary Results

Descriptive statistics were employed to explore the characteristics of the real sample using JMP^®^ Pro version 17. The mean number of pigs was 94, with a large standard deviation of 63.4. Initially, attempts were undertaken to obtain the exact solution of the TOP for the abattoir using the mathematical formulation presented in Section 2. As anticipated, it proved impossible to obtain an optimal solution even after running the model for 24 h. However, to validate the correctness of the mathematical model, small instances with 10 nodes were successfully solved.

Afterward, we solved the same problem using Algorithm 1 presented in Section 4.2. This solution is considered the base case for the scenario analysis. The results of the base case yielded a reward of 17,119 pigs slaughtered with a transportation cost (time) of 265.72 h, covering 91 routes. Solving the problem with different truck load capacities produced the results shown in Table 2. It is noteworthy that the costs are similar, as well as the number of routes. A simple calculation of mean travel time (cost) per route shows that it ranges from 2.920 to 3.007 h per route. However, the reward changes, increasing the number of pigs delivered to the abattoir as the truck load capacity increases.

### 5.2. Scenario Analysis

**Figure A3:**
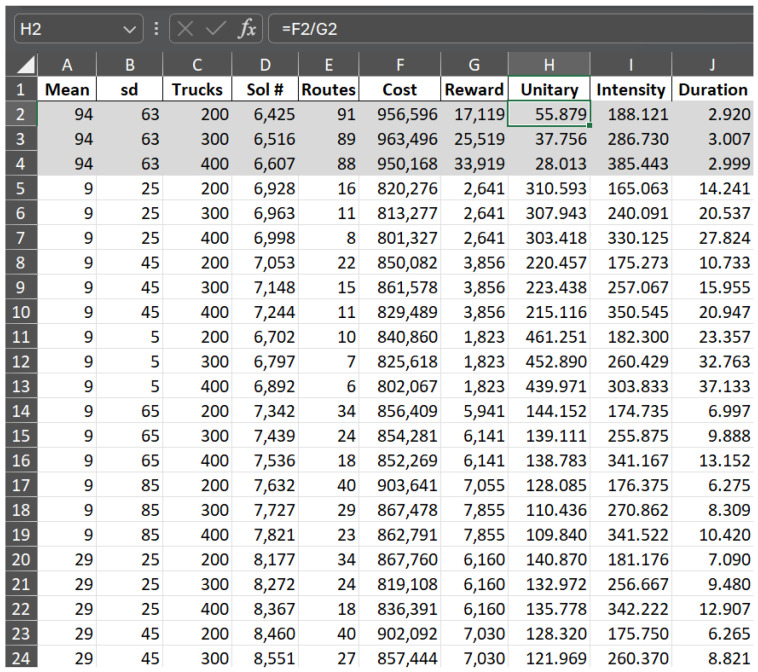
Results summary for each solution.

**Figure A4:**
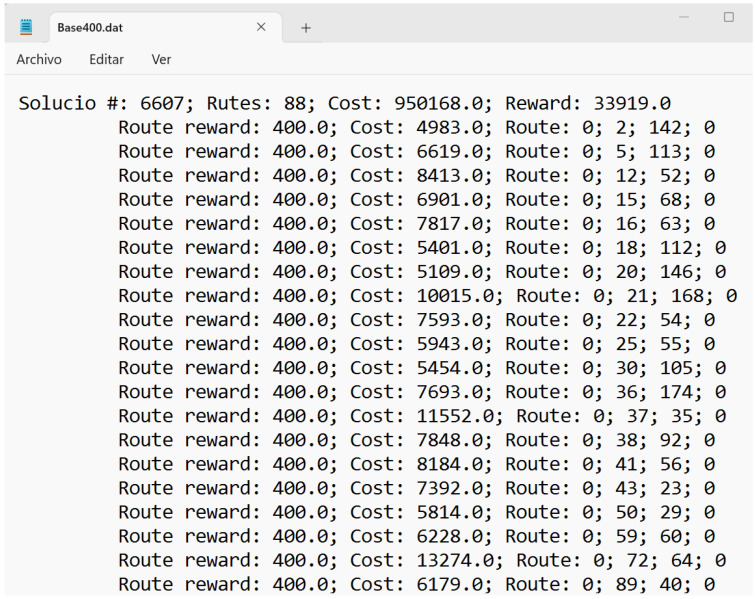
Routes recorded for each solution.

A summary of the rewards and costs is presented in Table 3 and Table 4. These results correspond to the average calculated for the three different solutions obtained with different trucks, i.e., different load capacities. The results summarize the outcome of the different scenarios generated according to the method presented in Section 4.4. This summary provides an overview of the average reward and cost obtained across different scenarios, offering insights into the overall performance of the solutions generated. Detailed sample outputs are shown in Figure A3 and Figure A4, included in Appendix A.1.

Table 3 illustrates that, on average, a higher number of pigs available on farms leads to a greater number of pigs being delivered to the abattoir. However, the variability observed in the number of pigs available (σ) does not consistently correspond to a higher number of pigs delivered to the abattoir. This discrepancy arises from the fact that random numbers are generated within the interval [0,200], and high variability may result in fewer pigs available for slaughter. This observation aligns with the trends observed in the associated cost (see Table 4) as a higher number of pigs delivered implies a higher cost and vice versa.

A summary of the correlation observed among standard deviation, truck capacity, and output variables (Table 5) helps to understand the positive and negative correlations among them. Notice the strong positive correlations between the trucks and intensity (i.e., pigs per route). This is reasonable since, the higher the truck capacity, the more pigs are transported.

The variable Sol# serves as a rough indicator of the algorithmic performance, representing the number of iterations in which the best solution is found. It is positively correlated with Routes, Cost, and Reward. This suggests that higher computational time tends to yield better results in terms of Reward, Cost, and Routes. Another notable correlation is observed between Routes and Cost, indicating that a higher number of routes requires more time to cover them. Among the auxiliary variables, Duration (hours per route) is highly correlated with Unitary (hours per pig). The negative correlations are of lesser intensity compared to the positive ones. The lowest value corresponds to Unitary (hours per pig) versus Reward (pigs), indicating that, the higher the transportation time per pig delivered to the abattoir, the fewer pigs are delivered.

### 5.3. Exploring the Results on Routes

**Figure 3:**
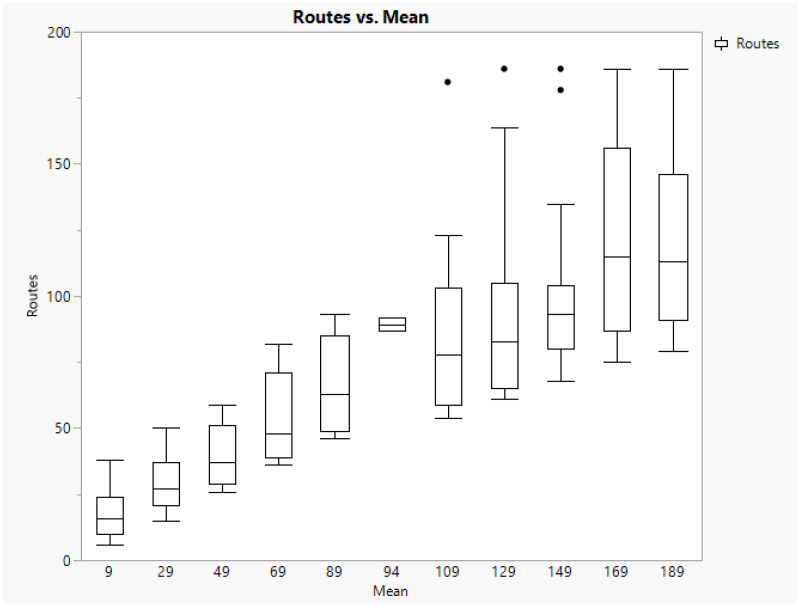
Boxplot of routes by mean number of pigs per farm.

After conducting the scenario analysis, it becomes evident that the primary outputs revolve around the number of routes, the cost, and the reward. The distribution of Routes in Figure 3 illustrates how the number of routes increases as the number of pigs to deliver per farm rises. Simultaneously, this increment contributes to higher variability observed in the boxplot as the mean number of pigs on farms increases. The boxplot with a mean of 94 pigs per farm corresponds to the base case (real-life instance), while the rest correspond to randomly generated instances.

One could think that the variability observed in Figure 3 could be attributed to the variance in the random generation of scenarios, specifically the number of pigs in fattening farms. However, for each mean, five different standard deviations are considered. Hence, the mean number of Routes for different standard deviations (SD) indicates that higher variability leads to less dispersion in the number of Routes. Upon investigating this issue, we concluded that a large standard deviation around the mean number of pigs in farms generates a variety of random values, which makes truck loading easier to complete. The different numbers of pigs available among different farms complement each other better to fill a truck, resulting in a lower cost as well.

In extreme scenarios, another noteworthy aspect is the number of farms visited per route, which was one or two for an average of one hundred pigs per farm or more. For biosecurity reasons [21], it is recommended that no more than two farms belonging to the same company be visited by a truck. Scenarios with a low average number of pigs to be collected per farm (e.g., nine) involved up to twenty farms visited. Although this number of visits is not realistic, it is worth noting that the average number of pigs per farm is not realistic either.

### 5.4. Exploring Results on Truck Capacity

**Figure 4:**
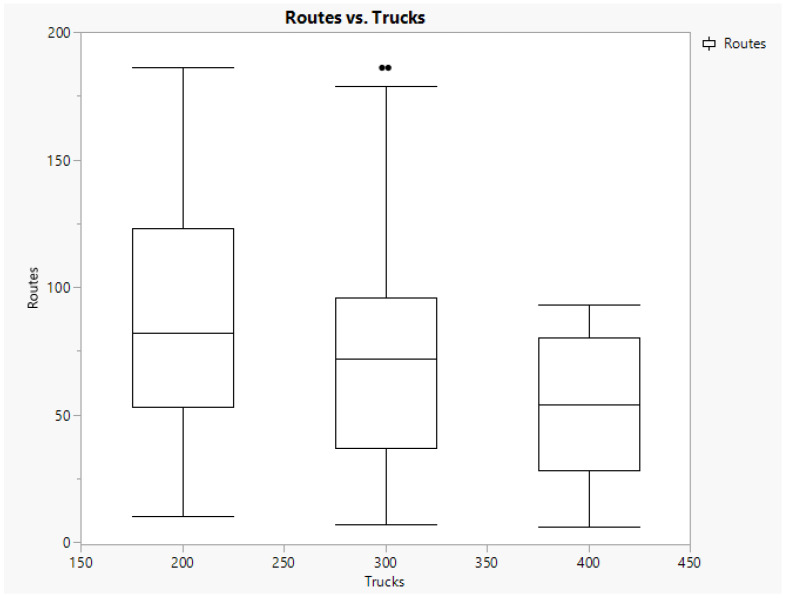
Boxplot of routes by truck load capacity.

We considered different load capacities for trucks to explore their effect on the number of routes to be implemented. This information is shown in Figure 4. As expected, higher load capacity implies fewer routes to cover and less variability. Figure 4 demonstrates a clear decrement in the average number of routes when truck capacity is increased. This effect may imply an interesting reduction in greenhouse gas emissions.

**Figure 5:**
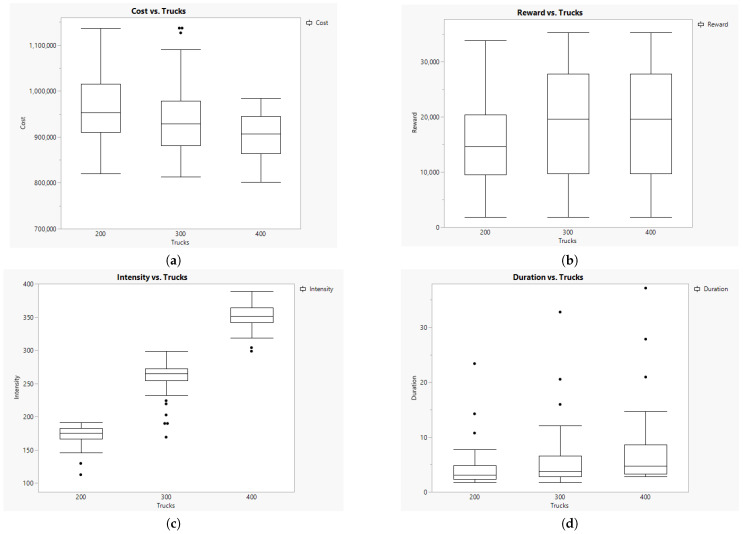
Results of Cost (**a**), Reward (**b**), Intensity (**c**), and Duration (**d**) per Truck capacity.

Other outputs are also affected by the load capacity of trucks (see Figure 5). For instance, cost is reduced when capacity is higher (Figure 5a), while the impact on reward is more limited (Figure 5b). This might be due to the fact that the total capacity of the fleet surpasses the number of pigs in farms to collect. Accordingly, the intensity of completing truck capacity increases with truck capacity (Figure 5c). At the same time, trip times represented by duration increase slightly because more truck capacity allows the company to plan longer routes (Figure 5d), thus implying less cost per truck and more reward capacity.

### 5.5. The Impact of the Number of Pigs to Collect

**Figure 6:**
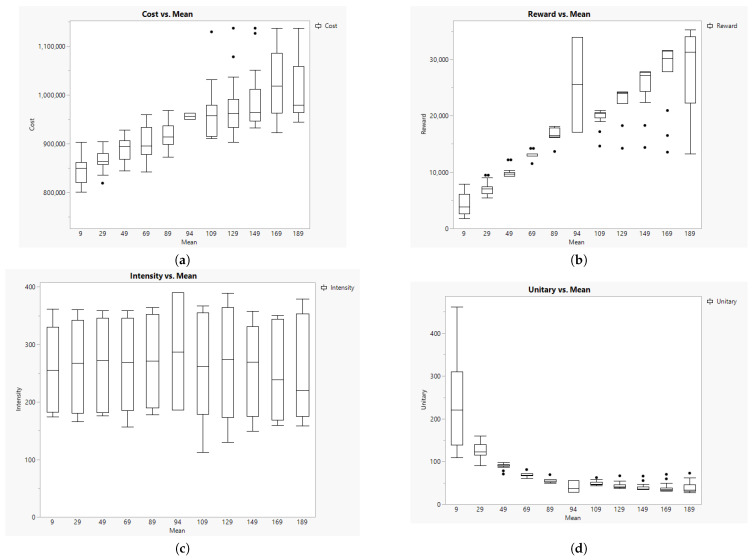
Results of Cost (**a**), Reward (**b**), Intensity (**c**), and Unitary (**d**) per mean number of pigs to collect per farm.

The number of pigs to be delivered to the abattoir is a crucial element in solving the TOP. A low mean number of pigs available for slaughtering may imply longer routes to fulfill the truck capacity, while more pigs may limit the flexibility to complete the truck load. Figure 6 summarizes the impact of different average numbers of pigs per farm available to be sent to the abattoir. Figure 6a represents the increasing cost when the average number of animals increases. Logically, if there are more pigs in the system, then more trips are required to the abattoir. This is confirmed with Figure 6b, showing that the number of pigs generating rewards depends on the mean value considered.

Another observation is that the intensity is not affected by the number of pigs to be collected (Figure 6c) because we only have three different load capacities. However, the trip time per pig (unitary) is reduced as the mean number of pigs per farm increases (Figure 6d). This result suggests better efficiency in transportation when more animals are available to load in trucks.

## 6. Conclusions

According to the objective of this study, we have successfully described the problem of marketing fattened pigs to the abattoir from the abattoir’s perspective by employing a TOP model. This TOP model has been formulated as a mixed integer linear problem. Solving this problem for large-sized instances in short computing times is unfeasible. Hence, an approximate method has been employed to solve a real-life instance of the problem. The solution algorithm proposed is based on a combination of the PJS heuristic and biased randomized techniques.

Regarding the managerial nature of the problem, we observed that the rewards from the pigs were more attractive than a mere reduction in the transportation cost. Another important aspect to observe was the truck capacity, which limited the number of farms to visit or the number of pigs to deliver, making it easier to resolve the problem when the variability was higher.

The proposed model performed well and is practical for planning the collecting routes of fattened pigs to be delivered to the abattoir. However, there are technical aspects relying on the uncertainty of the rewards or the interest in outsourcing the transportation that may deserve more investigation. In addition, the postponement of a visit to a farm and the collection of pigs may have a cost or a reward depending on the growth, weight, and sales price of the pigs. For that reason, in the near future, we plan to extend the model introduced here to a multi-period one.

## Figures and Tables

**Table 1:** Auxiliary outcomes calculated from instance results.

	Calculation	Explanation	Units
Unitary	cost/reward	Transportation time per pig	hours/pig
Intensity	rewards/routes	Pigs per route	No. pigs/route
Duration	cost/routes	Transportation time per route	hours/route

**Table 2:** Main results of the base case.

Truck Load	Cost (h)	Reward (€)	Routes (*n*)
200	265.72	17,119	91
300	267.64	25,519	89
400	263.94	33,919	88

**Table 3:** Reward per scenario (μ,σ) expressed in number of pigs transported.

	σ = 25	σ	σ = 45	σ = 65	σ = 85
μ = 9	1823	2641	3856	6074	7588
μ = 29	5407	6160	7030	7357	9321
μ = 49	9324	9524	10,001	9544	11,572
μ = 69	12,892	13,240	13,244	13,869	12,439
μ = 89	16,577	16,551	17,708	17,457	15,390
μ = 109	20,403	20,538	19,780	19,703	17,949
μ = 129	24,126	23,939	23,693	21,576	19,555
μ = 149	27,755	27,586	25,961	23,744	21,034
μ = 169	31,486	30,352	27,056	25,692	23,136
μ = 189	34,790	30,110	27,624	26,136	24,843

**Table 4:** Transportation cost per scenario (μ,σ) expressed in hours.

	σ = 5	σ = 25	σ = 45	σ = 65	σ = 85
μ = 9	228.6	225.5	235.3	237.3	243.9
μ = 29	239.2	233.6	242.9	242.2	244.7
μ = 49	242.5	246.7	249.0	247.6	248.9
μ = 69	244.1	254.5	254.9	250.2	249.3
μ = 89	256.4	258.4	252.8	256.0	254.5
μ = 109	282.2	270.8	263.6	266.1	254.8
μ = 129	278.3	277.3	273.9	268.5	256.4
μ = 149	285.4	282.6	277.1	267.6	261.5
μ = 169	301.7	290.7	280.2	272.6	267.5
μ = 189	297.1	285.7	277.0	277.2	275.2

**Table 5:** Correlation among output variables of scenarios.

	SD	Trucks	Sol#	Routes	Cost	Reward	Unitary	Intensity	Duration
**SD**	1.0000	−0.0000	0.0738	−0.1633	−0.1540	−0.0718	−0.1756	0.0756	−0.1581
**Trucks**	−0.0000	1.0000	0.0188	−0.3545	−0.3497	0.1597	−0.0692	0.9643	0.2206
**Sol#**	0.0738	0.0188	1.0000	0.7346	0.7112	0.8391	−0.6595	−0.0328	−0.6483
**Routes**	−0.1633	−0.3545	0.7346	1.0000	0.9795	0.7919	−0.5668	−0.4534	−0.6443
**Cost**	−0.1540	−0.3497	0.7112	0.9575	1.0000	0.7643	−0.5625	−0.4527	−0.6416
**Reward**	−0.0718	0.1597	0.8391	0.7919	0.7643	1.0000	−0.7045	0.1044	−0.6662
**Unitary**	−0.1756	−0.0692	−0.6595	−0.5668	−0.5625	−0.7045	1.0000	−0.0832	0.9323
**Intensity**	0.0756	0.9643	−0.0328	−0.4534	−0.4527	0.1044	−0.0832	1.0000	0.1974
**Duration**	−0.1581	0.2206	−0.6483	−0.6443	−0.6416	−0.6662	0.9323	0.1974	1.0000

**Figure A1:**
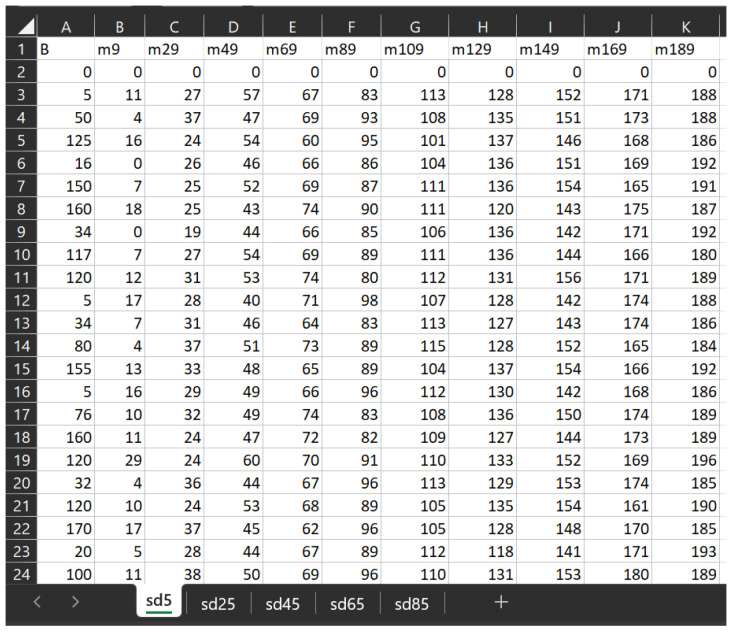
Number of pigs to collect generated randomly for each farm.

**Figure A2:**
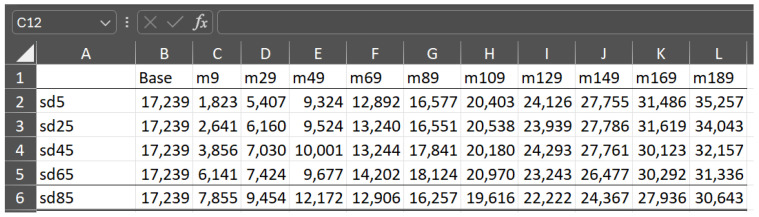
Total number of pigs to deliver by scenario.

**Figure A5:**
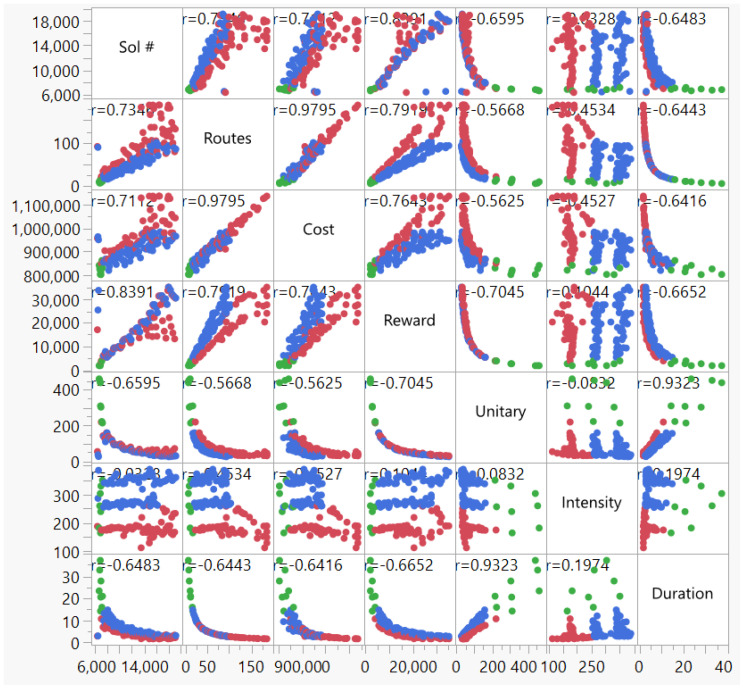
Multivariate correlations (the same color indicates the same cluster).

## Data Availability

Restrictions apply to the availability of these data.

## Abbreviations

The following abbreviations are used in this manuscript:

AIAO	All-In-All-Out
DSS	Decision Support System
IoT	Internet of Things
PJS heuristic	Panadero–Juan Savings heuristic
PSC	Pig Supply Chain
TOP	Team Orienteering Problem
UAV	Unmanned Aerial Vehicle
VRP	Vehicle Routing Problem

## Appendix A

### Appendix A.1

The specific random numbers generated to represent the number of pigs available at each fattening farm and the abattoir for each instance are shown in Figure A1. In particular, we observe the values generated for each node with the mean represented in the head of the column (e.g., m9, m10, …, m189), and each sheet represents a different standard deviation (e.g., sd5, sd10, …, sd85). The first column corresponds to the base case. Therefore, we can deduce that there are 50×186 random values generated according to 50 different normal distributions.

This approach enables the observation of different distributions of pigs among the farms. From these data, the total number of pigs available to collect in a given week can be computed, as shown in Figure A2.

Figure A3 displays an example of the main output variables recorded for each scenario in an Excel file. These variables include the number of solutions explored, the number of routes to cover in the solution, transportation cost (originally recorded in seconds), the number of pigs collected (reward), transportation time per pig, pigs transported per route, and transportation time per route. This comprehensive recording allows for detailed analysis and comparison of different scenarios.

An additional file contained detailed information about each solution, including the specific routes, nodes visited in each route, the load of animals, and the cost associated with each truck. This level of detail, illustrated in Figure A4, provides a comprehensive understanding of the logistics involved in transporting pigs from farms to the abattoir.

The correlations among variables are depicted in a multivariate plot in Figure A5. This visualization provides a clear understanding of the relationships between different output variables, such as the strong correlation observed between cost and routes.
